# *Pseudomonas aeruginosa *Cystic Fibrosis isolates of similar RAPD genotype exhibit diversity in biofilm forming ability *in vitro*

**DOI:** 10.1186/1471-2180-10-38

**Published:** 2010-02-08

**Authors:** Elena Deligianni, Sally Pattison, Daniel Berrar, Nigel G Ternan, Richard W Haylock, John E Moore, Stuart J Elborn, James SG Dooley

**Affiliations:** 1 Infection and Immunity Research Group, School of Biomedical Sciences, University of Ulster, Cromore Road, Coleraine, BT52 1SA, Northern Ireland, UK; 2 School of Medicine, Dentistry and Biomedical Sciences, Queen's University of Belfast, 73 University Road, Belfast, BT7 1NN, Northern Ireland, UK; 3 Systems Biology Research Group, School of Biomedical Sciences, University of Ulster, Road, Coleraine, BT52 1SA, Northern Ireland, UK; 4 Northern Ireland Public Health Laboratory Service, Department of Bacteriology, Belfast City Hospital, Belfast, BT9 7AD, Northern Ireland, UK

## Abstract

**Background:**

*Pseudomonas aeruginosa *is considered to grow in a biofilm in cystic fibrosis (CF) chronic lung infections. Bacterial cell motility is one of the main factors that have been connected with *P. aeruginosa *adherence to both biotic and abiotic surfaces. In this investigation, we employed molecular and microscopic methods to determine the presence or absence of motility in *P. aeruginosa *CF isolates, and statistically correlated this with their biofilm forming ability *in vitro*.

**Results:**

Our investigations revealed a wide diversity in the production, architecture and control of biofilm formation. Of 96 isolates, 49% possessed swimming motility, 27% twitching and 52% swarming motility, while 47% were non-motile. Microtitre plate assays for biofilm formation showed a range of biofilm formation ability from biofilm deficient phenotypes to those that formed very thick biofilms. A comparison of the motility and adherence properties of individual strains demonstrated that the presence of swimming and twitching motility positively affected biofilm biomass. Crucially, however, motility was not an absolute requirement for biofilm formation, as 30 non-motile isolates actually formed thick biofilms, and three motile isolates that had both flagella and type IV pili attached only weakly. In addition, CLSM analysis showed that biofilm-forming strains of *P. aeruginosa *were in fact capable of entrapping non-biofilm forming strains, such that these 'non-biofilm forming' cells could be observed as part of the mature biofilm architecture.

**Conclusions:**

Clinical isolates that do not produce biofilms in the laboratory must have the ability to survive in the patient lung. We propose that a synergy exists between isolates *in vivo*, which allows "non biofilm-forming" isolates to be incorporated into the biofilm. Therefore, there is the potential for strains that are apparently non-biofilm forming *in vitro *to participate in biofilm-mediated pathogenesis in the CF lung.

## Background

A biofilm is defined as a bacterial population in which the cells adhere to each other and to surfaces or interfaces with architectural complexity [1]. The role of biofilms in many infectious diseases including urinary tract infections [2], periodontitis [3], ophthalmic infections [4], and chronic diseases such as cystic fibrosis (CF) [5], has been demonstrated and they are thus of clinical concern. Biofilms exhibit increased resistance to antimicrobial agents, due to production of extracellular polymeric substances, high concentrations in the biofilm of enzymes such as β-lactamases due to higher cell density, slower cellular metabolic rates as a response to nutrient limitation and the presence of persistent cells [3,6-8].

The bacterial pathogen *P. aeruginosa *is capable of adhering to a variety of epithelial cells and this is believed to be the critical step in colonisation of the lung in CF. When sputum samples from CF patients were examined, *P. aeruginosa *predominated in aggregates, being encased in the characteristic extracellular matrix of biofilm thriving bacteria [9-11]. The early-infecting *P. aeruginosa *strains of the CF lung typically resemble those found in the environment, being non-mucoid, fast growing and relatively susceptible to antibiotics [12]. During chronic infection, however, the bacteria acclimatise to the airway environment of the CF patient via considerable genetic adaptation and the accumulation of loss-of-function mutations. Mutation in the *mucA *gene, for example, causes a transition from the non-mucoid to the mucoid, alginate-overproducing phenotype [13]. Other phenotypic changes include the loss of flagella or pilus mediated motility, the loss of O-antigen components of the lipopolysaccharide (LPS), appearance of auxotrophic variants and loss of pyocyanin production, as well as the emergence of multiply antibiotic resistant strains [8,11,14-16]. This phenotypic transition during chronic infection probably reflects an adaptive behaviour that enables the *P. aeruginosa *isolates to survive in the hostile environment of the CF lung [17-19].

Various studies have addressed the importance of bacterial motility, both as a means of initiating contact with an abiotic surface and in biofilm formation and development [20-22]. *P. aeruginosa *is capable of three types of motility. Twitching motility is mediated by type IV pili on solid substrates [12], whilst swimming motility and swarming motility are both mediated by the flagellum in aqueous environments. A switch from swimming to swarming motility is believed to occur in semisolid environments (e.g. agar or mucus) [23]. Flagella-mediated motility serves to bring cells into close proximity with surfaces thereby overcoming repulsive forces between the bacterium and the surface to which it will attach [24]. It has been suggested that the flagellum also plays a direct role as an adhesin [14] and indeed Sauer *et al*. [16,25], observed a significant decrease in attachment efficiency in non-flagellated *P. aeruginosa *mutants compared to the wild type. Twitching motility is a form of surface translocation that is mediated by type IV pili, which are involved in biofilm architecture and are responsible for the formation of microcolonies in biofilms [15,21,26].

It has been hypothesised that biofilm formation initially requires flagella-dependent association and binding to a surface to allow formation of a single cell monolayer. Individual cells of this monolayer then conglomerate into a number of microcolonies through twitching motility via type IV pili. Once attached and manifesting twitching motility, *P. aeruginosa *can then form fully mature biofilm structures [8,21]. Notably, cell motility varies during the different developmental stages and ceases after irreversible attachment, implying the loss of flagella in biofilm bacteria [16], a theory supported by microarray analyses that showed that flagella and type IV pili genes were downregulated in biofilm cells compared to planktonic cells [27]. In contrast, Klausen *et al*. [28] reported that flagella and type IV pili were not necessary for initial attachment or biofilm formation, but they did have roles in shaping *P. aeruginosa *biofilms: whilst both wild type PAO1 and flagella^-^/pili ^- ^mutants formed undifferentiated biofilms consisting of small microcolonies in the initial stages, the mature biofilms were structurally very different.

It is clear, therefore, that there is a large amount of information about the role of motility in biofilm development, but its contribution to the infection process is not fully clarified. However the adaptations that bacteria undergo in the CF environment are likely to induce alterations in the biofilm phenotype. In the present work, RAPD profiling was coupled with biofilm formation and motility studies *in vitro *to gain insight into how motility might be correlated with single or multistrain biofilm formation in CF isolates.

## Methods

### Chemicals

All chemicals, of analar grade or better were obtained from Sigma-Aldrich Chemical Co., Poole, UK, unless otherwise stated. All agars and broths were obtained from Oxoid, UK, except where stated.

### Bacterial isolates

Ninety-six *Pseudomonas aeruginosa *isolates were cultured from sputum samples taken from 13 children known to be infected only with *P. aeruginosa*, who were attending the CF clinic in Belfast City Hospital, N. Ireland at the same time (Andrienne Shaw, *pers*. *Comm*. 2003). Isolates were chosen based on their colony morphology on *Pseudomonas *isolation agar. All isolates were initially confirmed as *P. aeruginosa *using both the API20 NE identification system (BioMerieux, France) and by subsequent amplification of the *P. aeruginosa-*specific *OprL *gene [17]. Isolates were deemed mucoid or non-mucoid by visual inspection on agar and the collection was stored at -70°C on Protect cryobeads (Technical Service Consultants, Heywood, U.K.).

### Arbitrarily primed-PCR (AP-PCR)

Genotyping of the *P. aeruginosa *collection used two arbitrary primers, 10514 and 14306 (Table 1), as described by Kersulyte *et al*. [29]. Phylogenetic trees were constructed using the Gelcompar II software (Applied Maths BVVBA, Keistraat 120, 9830 Saint-Martens-Latem, Belgium). The cluster algorithm used was UPGMA and DICE with an optimisation value of 0.5% and a tolerance of 1%. Gelcompar II software was used to generate profiles.

**Table 1:** Primers used in this study.

f	Primer sequence	Application	Reference
PAL1	5'-ATGGAAATGCTGAAATTCGGC-3'	Amplification of the *OprL *gene	De Vos *et al*. 1997
PAL2	5'-CTTCTTCAGCTCGACGCGACG'-3	Amplification of the *OprL *gene	De Vos *et al*. 1997
10514	5'-TGGTGGCCTCGAGCAAGAGAACGG-3'	RAPD analysis	Kersulyte *et al*. 1995
14306	5'-GGTTGGGTGAGAATTGC-3'	RAPD analysis	Kersulyte *et al*. 1995
pilA	5'-ATG AAA GCT CAA AAA GGC TTT ACC TTG AT-3'	Identification of *pilA*	Kus *et al*. 2004
pilB	5'-TCC AGC AGC ATC TTG TTG ACG AA-3'	Identification of *pilA*	Kus *et al*. 2004
pilB2	5'-TGT TCA GGT CGC AAT AGG C-3'	Identification of *pilA*	Kus *et al*. 2004
pilB3Rev	5'-CGG AGA TGC CTA CAA AGA GC	Identification of *pilA*	This study
nadCFor	5'-CAG AAG TAC GCG GTC ACC TG	Identification of *pilA*	This study
tRNA^Thr^	5'-CGA ATG AGC TGC TCT ACC GAC AGA GCT-3'	Identification of *pilA*	Kus *et al*. 2004
fliCFor	5'-GGC CTG CAG ATC NCC AA	Identification of *fliC*	Winstanley *et al*. 1996
fliCRev	5'-GGC AGC TGG TTN GCC TG	Identification of *fliC*	Winstanley *et al*. 1996
fliCRev2	5'-TTA GCGCAG CAG GCT CAG	Identification of *fliC*	This study
fliCFor3	5'-ATG GCC TTG ACC GTC AAC ACC	cloning of *fliC*	This study
fliCFor2	-ATG GCC CTT ACA GTC AAC ACG	cloning of *fliC*	This study
SeqU19	5'-GGT TTT CCC AGT CAC GAC G	sequencing of all cloned *pilA *and *fliC*	This study
SeqT7	5'-CTA ATA CGA CTC ACT ATA GGG	sequencing of all cloned *pilA *and *fliC*	This study
pre-pilA	5'-GCG TTT GAA AGG TTG GCA TGC	sequencing of all cloned *pilA*	This study
transrev	5' CAG CAT AAC TGG ACT GAT TTC AG-3'	To check successful conjugation of the mini-Tn*7 *anneals to the inserted DNA	Koch *et al*. 2001
transfor	5'-AAT CTG GCC AAG TCG GTG AC-3'	To check successful conjugation of the mini-Tn*7*, anneals to the 3'end of *glmS*	Koch *et al*. 2001

### Motility assays

#### (i) swimming

Cells were transferred to semi-solid agar medium (10 g l^-1 ^tryptone, 5 g l^-1 ^NaCl, and 0.3% (wt/vol) DNA grade agarose (BDH Ltd., UK) using a sterile toothpick. The swimming zones were measured after 48 h incubation at 37°C. Swimming motility was also confirmed by light microscopy.

#### (ii) swarming

The medium used for this assay consisted of 0.5% Nutrient broth, 5 g l^-1 ^glucose and 0.5% Bacto-Agar (Difco). Plates for swarming motility assays were inoculated with a 5 μl aliquot from an overnight culture in LB broth, onto the top of the agar and incubated at 37°C for 48 h.

#### (iii) twitching

The plates for twitching motility contained LB broth solidified with 1.2% bacteriological agar. The plates were stab inoculated with a sharp toothpick to the bottom of a Petri dish from an overnight culture grown on LB Agar at 37°C. The plates were incubated at 37°C overnight and the clear zone at the agar/Petri dish interface was measured as per Harunur-Rashid and Kornberg [30] followed by staining with coomassie brilliant blue G250 (0.5% (w/v) in 25% (v/v) isopropanol/10% (v/v) acetic acid) for 30 min to increase contrast. All motility assays were performed in triplicate.

**Detection of *pilA *and *fliC *genes **was confirmed as described by Kus *et al*. [31] with modifications in the primers as shown in Table 1. ***PilA ***genes of isolates 1, 40 and 48 were amplified with the primer set pilB2 and tRNA^Thr^, and for isolate 72, the primer set pilA and tRNA^Thr^. ***FilC ***genes of isolates 1 and 72 were amplified with primers fliCFor3 and fliCRev2 [32], and for isolates 40, 41 and 48 the primer set fliCFor2 and fliCRev2. The resultant amplicons were ligated into a pT*7*Blue-2 cloning vector and transformed into NovaBlue Singles using a Perfectly Blunt Cloning Kit (Novagen). Plasmid DNA was extracted from broth cultures using a Rapid Plasmid Miniprep Kit (Qiagen) and the inserts sequenced. Primers SeqU19, SeqT7 and pre-pilA were used in the sequencing of all cloned *pilA *genes. In addition, clones from isolates 1, 40 and 48 required use of primer pilB2 while isolate 72 required the primer pilA. Primers SeqT7 and SeqU19 were used to sequence the cloned *fliC *genes from all four isolates. The sequences for isolates 1, 40, 41 and 48 have been deposited in GenBank. For the *fliC *gene the accession numbers are EF418192, EF418193, EF418194, and EF418195 respectively while for the *pilA *gene EF418188, EF418189, EF418190 and EF418191, respectively).

**Gfp tagging of *P. aeruginosa *isolates **was carried out by mobilising the pBK-miniTn*7*-*gfp*3 and pUX-BF13 plasmids (Table 2) as per Koch *et al*. [13]. Insertion was confirmed by PCR using transrev/transfor primers (Table 1) giving a 150 bp amplicon.

**Table 2:** Strains and plasmids used in this study.

Strain/plasmids	Genotype/phenotype	Source/reference
***E. coli*** ***E coli *JM109**	End1 recA1 gyrA96 this hsdR17(r_k_^-^m_k_^+^) relA1 supE44 Δlac-proAB (F' traD36 proAB lacI^q^ZΔAM15)	Promega
***P. aeruginosa*** **ATCC 15442**		Centre for Biofilm Engineering, Montana
**Plasmids**		
pRK2013	ColE1-Tra(RK2)^+^Km^r^	Figurski & Helinski, (1979) [47]
pUX-BF13	R6 K replicon -based helper plasmid providing the Tn7 transposition function in trans. Ap^r^, mob^+^	Bao *et al*. (1991) [48]
pBK-miniTn7-gfp3	pUC19 based delivery plasmid or miniTn7-gfp3. Km^r^, Ap^r^, Cm^r^, Sm^r^, mob^+^	Koch *et al*. (2001)

### Microtitre plate assay for assessment of biofilm formation

*P. aeruginosa *strains were grown to an attenuance (D_600 nm_) of 0.5 and diluted 100-fold with LB broth following which 100 μl aliquots were dispensed into triplicate microtitre plates which were incubated at 37°C. Three plates were sacrificed for analysis every 4 h and attenuance was measured at 600 nm with a BioTek FL600 microtitre plate reader against uninoculated LB broth; means and standard deviations were calculated. Biofilms were stained with 1% crystal violet, washed with deionised water and quantitated by adding 95% ethanol followed by measurement of the absorbance (OD 595 nm) as per Stepanovic *et al*. [33]. Strains with no change in O.D over the control were classified non-biofilm producers, weak- (up to a 2 fold change), moderate- (up to 4 fold change) or strong- (greater than 4 fold change) as per Strepanovic *et al*. [33] All tests were carried out in triplicate and the results were averaged. *P. aeruginosa *strain PAO1 was included as a positive control.

**Biofilms in a capillary flow reactor **were grown in glass capillary tubes of square cross sections under continuous flow conditions. The capillaries had a nominal inside dimension of 900 μm and a wall thickness of 170 ± 10 μm (Friedrich & Dimmock, Millville, N.J., USA). The flow cell apparatus consisted of a vented medium feed carboy (four litre capacity), a flow break, a filtered air entry, a peristaltic pump (Watson-Marlow), the capillary and flow cell holder, an inoculation port, and a waste carboy. The components were connected by silicone rubber tubing and were sterilised by autoclaving.

A culture of *gfp*-*P. aeruginosa *was grown in LB overnight at 37°C in a shaking incubator at 140 rpm. A 100 μl aliquot of this culture was used to inoculate 10 ml of sterile LB broth in a 250 ml conical flask to achieve good aeration and the culture was grown at 37°C with shaking at 200 rpm for 3 h. The tubing was clamped downstream of the inoculation port and the capillary flow system was inoculated with 300 μl of this fresh culture. The tubing was then clamped upstream of the glass tube and the system was allowed to stand without a flow for 19 h to allow the cells to attach to the glass capillary at 37°C. After initial attachment, the flow of medium (1/10 strength LB, to avoid blockage of the capillary due to excessive biomass production) was adjusted to a flow rate of 20 ml h^-1^.

**Bacterial staining of mixed biofilms **consisting of biofilm^+ ^and biofilm^- ^isolates, were stained with 300 μl of a 5 mg l^-1 ^rhodamine B (Kodak) solution in water. The stain solution was injected into the capillary reactor through the inoculation port and the cells allowed to stain for 5 min. Biofilms were subsequently observed by confocal scanning laser microscopy with excitation and emission wavelengths of 540 nm at 625 nm respectively for rhodamine B and 475 nm and 510 for GFP.

### Scanning Electron Microscopy (SEM)

Prior to SEM, samples were chemically fixed as follows: A 10 μl aliquot of an overnight culture, grown in LB broth at 37°C, with shaking at 140 rpm was placed in a round glass coverslip (10 mm diameter, Chance Proper Ltd., UK) with a 10 μl of fixative (3% glutaraldehyde in 0.1% sodium cacodylate, pH 7.3). The coverslips were previously coated with polylysine (Sigma-Aldrich) to assist adherence of bacterial cells. The cells were allowed to fix for 10 min following which the supernatant was discarded and excess fixative was added to the coverslip for 5 min followed by a final washing step with 0.1 M sodium cacodylate, pH 7.3. In order to dehydrate the bacteria the coverslips were successively placed for 10 min in each one of the following solutions: 30%, 50%, 70%, 90%, and 100% (twice) (v/v) acetone. The coverslips were then dried with a critical point drier and sputter coated with Au: Pt, 60:40 in argon (Polarow E5100). The slides were visualized with a JSM 840 SEM (JEOL Ltd., Herts, UK).

Light and Epifluorescence microscopy examination of *P. aeruginosa *cells was performed using a Nikon Eclipse E800 microscope equipped with 40 × and 60 × water objectives, differential interference contrast (DIC) polarizing filters and reflectance optics. For epifluorescence microscopy, the microscope was equipped with a 100 W Hg-vapour discharge lamp and fluorescent images were obtained using the following filters: B-2A blue excitation filter with excitation wavelength 470-490 nm, (Nikon) and a Red excitation filter: Cy5 HYQ (Nikon). Images were captured by a Micromax RTE/CCD-732-7 (Princeton Instruments, Trenton, NJ, USA) camera and MetaVue 5.0 software (Universal Imaging Co., Downingtown, PA, USA).

### CLSM and image analysis

Glass capillary flow reactors were inoculated with the GFP-*P. aeruginosa *isolates and biofilms in capillary flow reactors were observed using 40 × magnification lenses with a CLSM (Leica TCS-NT). CSLM image analysis software was Image Pro Plus, Version 3.00.00 (Media Cybernetics, Bethesda, MD, USA). Microscope images were analyzed by use of the line scan fiction of Metamorph image analysis software (Universal Imaging Co., Downingtown, PA, USA). For the depth profile, the interface between the biofilm and the glass wall was set to zero on a spatial axis. Stimulated fluorescence projections and vertical cross sections through the bacterial biofilms were generated with IMARIS (Bitplane AG) software package running on a Silicon Graphics Indigo 2 workstation.

**Statistical analysis **was performed in order to validate the effect of motility in *P. aeruginosa *biofilms. The isolates were divided into four groups based on their motility patterns: the first group (C1) consisted of isolates that both swim and twitch, the second (C2) of immotile isolates, the third (C3) of isolates that swim but do not twitch and the forth (C4) of isolates that twitch but do not swim. A one-way ANOVA was performed to test the null hypothesis that there were no differences in the mean motility of the four groups, followed by a Tukey's post-hoc to compare the individual groups' differences. Tukey's post-hoc calculates a 95%-confidence interval for the mean of each group and then substracts the means pair-wise i.e. C1 minus C2, C1 minus C3 etc. If the differences include 0 then the means are not significantly different. The results were verified by performing a two sample Ttest within pairs of groups and the obtained p-values for multiple testing corrected using Bonferroni correction. MiniTab was used for the statistical analysis.

### Statement of Ethical Approval

Research carried out in this study was approved by Health and Personal Social Services (HPSS) (Northern Ireland) REC 2, Reference No. 07/NIR02/39.

## Results

We examined a set of 96 clinical isolates of *Pseudomonas aeruginosa *for their ability to produce biofilm *in vitro *and we determined the relationship of bacterial motility to biofilm production within the set.

### Diversity in biofilm formation by *P. aeruginosa *CF isolates

We examined biofilm-forming ability in 96 well microtitre plates. Biofilm growth was observed as a ring of crystal violet-stained material formed at the air-liquid interface. We observed a wide variation in the quantity of biofilm biomass amongst the isolates tested (Table 3, column 3-5). A total of 31 isolates were characterised by weak adherence, 19 isolates by moderate adherence and 46 by strong adherence (A_595 _nm > 0.3). Among the strongly adherent isolates, differing levels of adherence were also observed, with A_595 _nm values ranging from 0.3-2.0. Neither the quantity of planktonic cell biomass produced in these cultures, nor the growth rate of the isolates, was correlated with the quantity of biofilm biomass produced: bacteria with doubling times of either 1 h or 5 h could both produce the same quantity of biofilm. Biofilm formation amongst the isolates also differed in the time of initial adhesion, with some isolates showing strong adherence whilst the planktonic bacterial population was still in the lag phase and the cell density low, while for others, adhesion commenced only when the planktonic culture was in the mid exponential phase (data not shown). A whole cell protein determination [34] carried out concomitantly with D_600 _nm measurements, confirmed that attenuance values were indeed due to planktonic cells and not due to alginate produced by them.

**Table 3:** Variability of biofim and motility phenotypes among a set of 96 clinical *Pseudomonas aeruginosa *isolates.

Genotypic profile^$^	Number of isolates in the given profile	biofilm			Motility		
		
		weak	moderate	strong	twitch	swim	swarm
1	7 (1)*	4	3		1		
2	1 (1)			1		1	
3	15 (4)	1	2	12			
4	5 (2)	1		4	5	5	5
5	1			1	1	1	1
6	2 (1)		2				
7	11 (3)	2	1	8	1	1	1
8	5 (2)		3	2			
9	4 (1)	1	1	3	4	3	
10	4 (1)			4	3	4	4
11	4 (1)	4				4	2
12	1	1				1	
13	1	1				1	1
14	2 (1)	1		1		1	
15	5 (1)			5	5	5	5
16	1	1					
17	11 (1)		1	10	5	9	5
18	2 (1)	1	1				
19	1	1					
20	2 (2)	1	1		1	1	
21	1			1			
22	10(1)	10			1	10	

* Number in brackets is number of patients from whom the strain derived.

^$ ^RAPD genotyping based upon primer 10514 and employing a cut off of 85% similarity.

**Figure 1:**
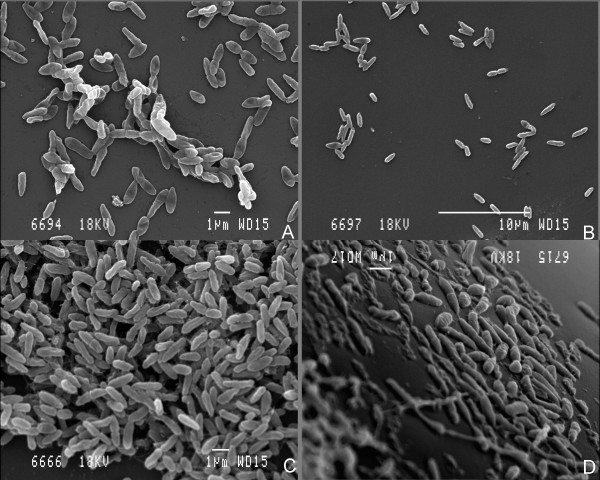
**Scanning electron microscopy images of *Pseudomonas aeruginosa *isolates attaching to glass surfaces**. Weakly adherent *P. aeruginosa *isolates formed a monolayer (B and D; isolate 80) while the moderate and strongly adherent isolates formed clumps of cells (A and C; isolate 17) when biofilms were grown on glass cover slips. Microbial attachment first presented as clumps of cells (A and B; 7 and 14 h respectively after inoculation) and as the biofilm matured the spaces between the clumps were covered with a cell lawn (C and D; 20 and 40 h respectively after inoculation).

In order to visualise the differences in attachment between strong and weak biofilm forming isolates, bacterial cells were allowed to attach to glass coverslips and subsequently visualized using SEM. Coverslips were immersed vertically in an inoculated culture and two coverslips were removed and fixed for SEM visualization at intervals. Diversity in isolate attachment onto the glass cover slip was observed, with the moderate and strongly adhering isolates from the microplate assay forming clumps of cells (e.g. isolate 17; Fig. 1a). Weakly adherent isolates attached as individual cells (e.g. isolate 80; Fig. 1b) however as both types of biofilm matured, the spaces between the clumps were filled with a cell lawn (Fig. 1c &1d).

Isolates previously characterised as weakly adherent did not form the characteristic biofilm structures, and we observed that relatively few cells were attached to the glass substrate and that biofilm formation was initiated only after the surrounding planktonic culture had reached stationary phase. At this point the cells were elongated, reaching up to 15 μm in length - a potential response to nutrient limitation also observed by other researchers.

### *P. aeruginosa *isolates from CF patients show diversity in motility phenotype

Having observed significant diversity in biofilm formation within the group of clinical isolates we then investigated isolate motility. Swimming motility was initially observed for 48 isolates (50%) with a migration zone of 7 - 40 mm (Table 3, column 7). Twitching motility was distinguished by the presence of an interstitial twitch zone formed by colony expansion. Isolates exhibiting twitching motility (Table 3, column 6) formed flat spreading colonies with a characteristic "rough" appearance and a twitching zone consisting of a very thin layer of cells observed as a halo around the colony. Isolates incapable of twitching formed small, smooth, flat colonies on the agar surface that remained at the inoculation point. Coomassie staining revealed a series of concentric rings in the twitching zone. When *P. aeruginosa *isolates were inoculated onto the surface of agar to assay swarming motility, 36 (37%) of the isolates (Table 3, column 8) formed characteristic swarming patterns consisting of branches or tentacles radiating from the inoculation point. Movement across the agar surface was rapid, with bacteria having colonised the entire surface of the plate within several hours after inoculation. A lack of twitching motility was not matched by an absence of swarming motility, but did seem to influence the pattern of colony translocation. When twitching motility was present, the swarm edge exhibited a higher bacterial cell concentration than the centre, while in non-twitching isolates, bacterial colonies were denser in the centre and surrounded by a thin film of translocated bacteria. Of the 96 isolates, a number of strains overlapped in terms of motility phenotype: 24 had flagellar and twitching motility, 27 had only twitching motility, 47 had only swarming motility and a total of 45 were non motile.

Given the complex phenotypic diversity of the clinical isolates based on direct observations we recognized the need for a rational approach to selecting the most appropriate isolates for further study. We adopted RAPD as a convenient and quick genotyping method that allowed us to characterise the heterogeneity in the group, using a cut off value of 85% similarity as a threshold to compare strains. Primer 10514 generated a total of 22 different profiles (Table 3), fifteen of which contained more than one isolate. Primer 10514-generated profiles were cross- referenced with those of primer 14306 and showed that similar profiles were generated with both primers.

We noted variations in surface attachment ability and in motility among strains and we selected strains based upon both genotypic and phenotypic characteristics, i.e. strains that represented similar RAPD groupings and also based upon the degree of biofilm production. Twenty genotypically distinct isolates were thus selected for further study (Table 4, column1).

**Table 4:** Correlation of the swimming phenotype of 20 selected clinical *Pseudomonas aeruginosa *isolates with the presence of *fliC *gene and correlation of the twitching phenotype with the presence of the *pilA *gene group.

Isolate	Swimming motility	*fliC *gene	Twitching motility	*pilA *gene group
1	+	+	+	II
3	+	+	+	II
7	-	+	-	I
17	+	+	+	I
26	+	+	+	I
29	-	+	-	I
30	-	+	-	I
33	+	+	-	I
38	+	+	+	I
40	+	+	+	I
41	+	+	-	-
46	-	+	-	I
48	-	+	-	I
54	+	+	-	-
55	+	+	-	-
64	+	+	+	II
72	-	+	-	V
80	-	+	-	I
85	-	+	-	I
94	-	+	-	I

### *P. aeruginosa *CF isolates exhibit a lack of correlation between motility phenotype and genotype

The observed phenotypic differences in twitching motility led us to consider whether non-twitching isolates were inherently non-motile or whether they possessed the capability to be motile but did not express it. Pilin alleles and associated gene(s) are located in a common chromosomal locus between the conserved *pilB *and *tRNA^Thr ^*genes [18]. The presence of various *tfp *accessory genes located upstream of *pilA *determines amplicon size, thus allowing the delineation of five TFP groups [18,31]. Seven twitching efficient and 13 twitching deficient isolates were selected (Table 4) and we determined whether or not *pilA*, the type IV pilus (TFP) gene responsible for the PilA structural protein, was present in the isolates.

Thirteen isolates yielded ~2.8 kbp amplicons with the pilB and tRNA^Thr ^primers [31], thus the majority of the CF isolates fell into TFP group I (*tfpO*). Amplicons of ~1.4 kb were produced with the same pilB and tRNA^Thr ^primer set from isolates 1, 3 and 64 placing them in TFP group II (*tfpY*). Six other primer combinations were tried with isolates 41, 54, 55 and 72, however a *pilA *amplicon was generated only from isolate 72 using primers pilA and tRNA^Thr^, showing that it belonged to TFP group V (*tfpZ*). Of the 17 isolates for which *pilA *presence was confirmed only 7 (41%) actually exhibited twitching motility, demonstrating that the presence of *pilA *alone does not secure motility. Representative amplicons were cloned and sequenced and subsequent alignments confirmed their categorisation into the groups described by Kus *et al*. [31]. The *fliC *structural gene was also detected in all 20 isolates (Table 4), however its presence, like that of *pil*A, did not guarantee swimming motility as 9 isolates (45%) did not swim. The presence of flagella in isolates was verified with SEM, while full length DNA sequences were obtained for *fliC *of isolates 1, 40, 41 (motile) and 48 (non motile).

### Statistical analysis shows that motility contributes to biofilm thickness but not to biofilm formation in our isolates

**Figure 2:**
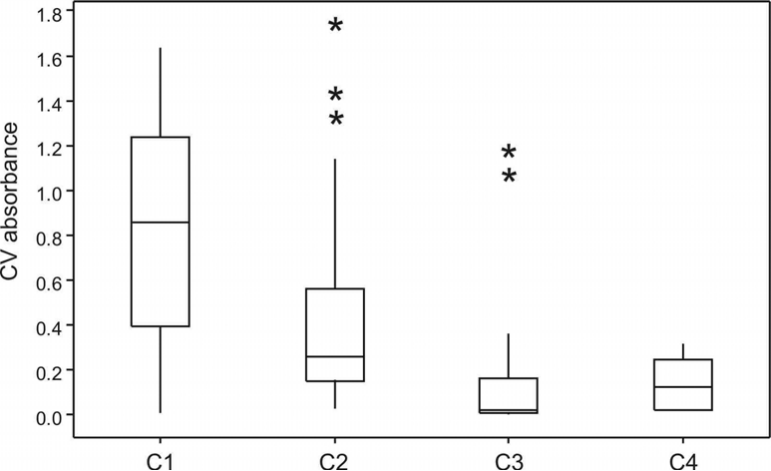
**Box-and-whiskers plots showing the impact of flagella/TFP on the biofilm**. *P. aeruginosa *isolates placed in four groups based on their motility properties. Based on the presence of flagella/TFP the groups were named as C1 (+/+), C2 (-/-), C3 (+/-), C4 (-/+). One-way ANOVA reveals a significant difference (*P *< 0.001) between the groups with respect to CV absorbance. (This difference can also be observed when the three outliers, marked by stars, in group C2 and the two outliers in group C3 are discarded from the analysis.) Tukey's post-hoc test revealed that the presence of both flagella/pili (group C1) contributes to a significantly higher biofilm biomass (as compared to groups C2-C4).

It has been reported in a number of studies [16,25,35,36] that motility is required for biofilm formation, whereas in contrast, Klausen *et al*. [28] reported that mutants deficient in pili and flagella showed no significant differences from wild type. In the current study, biofilm formation was not influenced by the presence of either flagella or type IV pili, since 45 isolates that formed either moderate or strong biofilms were deficient in twitching, swimming, and swarming motility. In contrast however, isolates 5, 6, and 61 (motile) exhibited very poor adhesion in microtitre plates. For the statistical analysis we started with the null hypothesis that motility does not affect biofilm formation and performed a one-way ANOVA that gave an *F-*value of 9.88, allowing rejection of the null hypothesis. At this point we could not say between which groups the difference was so we performed a Tukey's post-hoc test between all the possible group pairs. Group C1, as it was termed for the analysis, contained the highest percentage of strong biofilm forming isolates - 80% - while in groups C2 and C3 the percentage of strong biofilm forming isolates was only 40% and 33%, respectively (Fig. 2). The results revealed that C1 was different from C2, C3 and C4 but there was no difference among the C2, C3 and C4. The same conclusion was reached using a Ttest with correction for multiple testing. We concluded therefore that the combination of swimming and twitching motility has a positive contribution to biofilm biomass but is not absolutely necessary for the initiation of the process.

### Diversity in biofilm architecture among *P. aeruginosa *isolates

**Figure 3:**
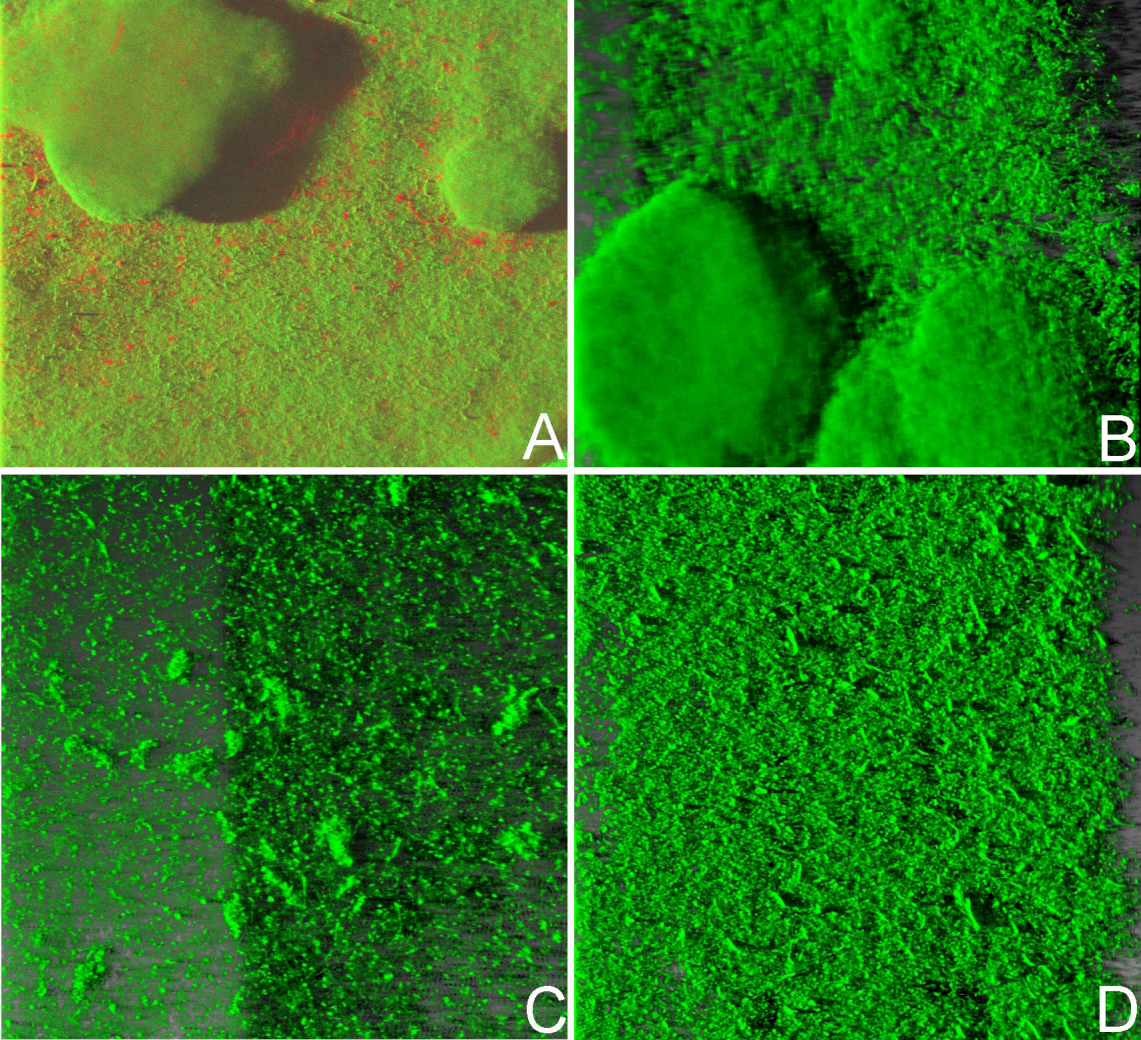
**CSLM images of GFP-tagged *Pseudomonas aeruginosa *biofilms in a glass capillary flow reactor 72 h post-inoculation, showing variation in biofilm structure**. (A) control strain *P. aeruginosa *ATCC15442; (B) *P. aeruginosa *CF isolate 17; (C) *P. aeruginosa *CF isolate 40 (D) *P. aeruginosa *CF isolate 41.

Having shown statistically that the isolates possessing both twitching and swimming motility produced greater biofilm biomass we set out to investigate the architecture of biofilms produced by members of this group. We *gfp *tagged 5 isolates exhibiting different motility/biofilm biomass combinations: 17 and 40 (twitch^+^, swim^+^, biofilm^+++^), 41 (twitch^-^, swim^+^, biofilm^+^), 55 and 80 (twitch^-^, swim^-^, biofilm^+^). The resulting *gfp*-tagged isolates had growth rates identical to those of the parental strains (data not shown). *P. aeruginosa *ATCC15442 was used as a control to ensure that reactor did not influence biofilm morphology and following staining with Syto9 and propidium iodide, characteristic mushroom-shaped biofilms of *P. aeruginosa *ATCC15442 were observed in a number of different reactors. Spatial biofilm distribution in the tagged strains was measured over time in a glass capillary flow reactor and images were acquired with CLSM at regular 12 h intervals at random positions in the flow chambers. Visual inspection revealed that the biofilm architecture of the *P. aeruginosa *CF isolates was significantly different from that of the ATCC control strain (Fig. 3). Among the isolates tested, 17, 40 and 41 gave biofilm growth while isolates 55 and 80 did not attach to the glass capillary. Isolates were observed as microcolonies on day 1 and formed a biofilm within 48 h of inoculation. They continued to grow until the 7^th ^day with a maximum thickness of 75 μm for isolates 17 and 40 and 145 μm for isolate 41. Isolate 17 formed a mushroom-shaped biofilm that appeared after 48 h of growth, while isolate 40 formed a flat biofilm with small hilly structures spatially distributed. The biofilm formed by isolate 41, was flat and was the thickest among the isolates. Although stains 55 and 80 showed weak attachment to microtitre dish wells, other than a transient superficial attachment at seven hours no attachment was observed from 12 hours onward in the glass capillary flow reactor. We observed that cell attachment proceeding to biofilm formation was dependent upon the attachment substrate.

### Entrapment of non-biofilm forming *P. aeruginosa *isolates by an already established biofilm

**Figure 4:**
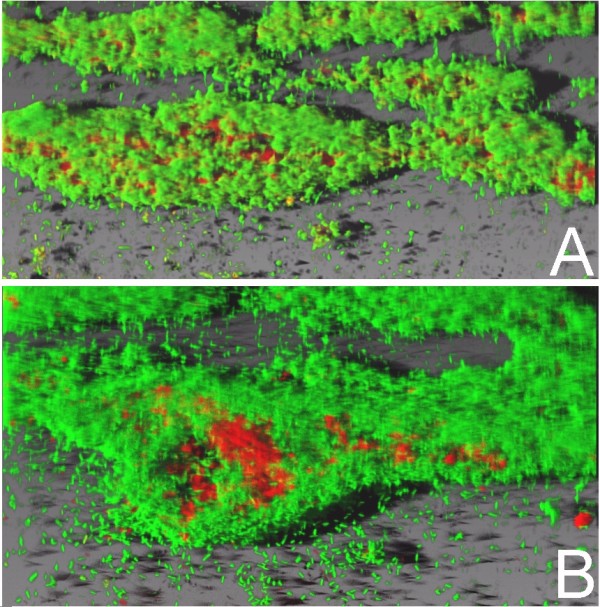
**CSLM images of mixed biofilm produced by *Pseudomonas aeruginosa *isolates *gfp*-17 (green) and isolate 80 (red) in a glass capillary flow reactor**. Isolate *gfp*-17 was allowed to establish a biofilm for 48 h and then isolate 80 was inoculated into the flow reactor. After 24 h incubation the mixed biofilm was stained and GFP and rhodamine B were excited at 488 nm and 567 nm respectively.

**Figure 5:**
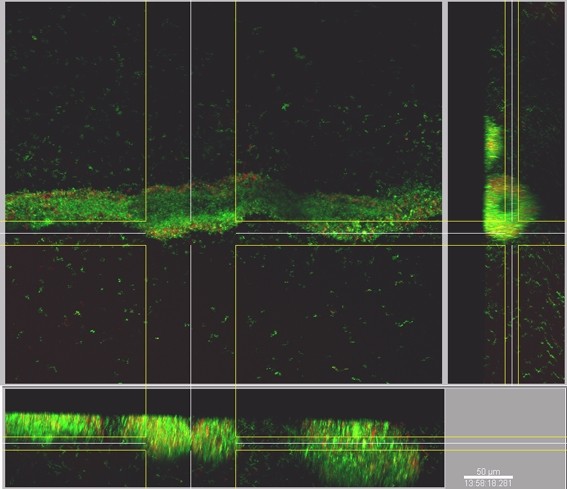
**Cross section of the mixed *Pseudomonas aeruginosa *biofilm**. Isolate *gfp*-17 was allowed to establish a biofilm for 48 h and then isolate 80 was inoculated into the flow reactor. After 24 h incubation the mixed biofilm was stained and GFP and rhodamine B were excited at 488 nm and 567 nm respectively. As can be seen from the cross section, isolate 80 became incorporated into the biofilm body and was not simply attached to the surface of the isolate *gfp*-17 biofilm.

We wished to test the hypothesis that the diversity of isolates derived from clinical samples is due to non-biofilm producing isolates interacting with biofilm producers. Glass capillary flow reactors were inoculated with the GFP-*P. aeruginosa *17 isolate and the biofilm formation was followed with CLSM. Following 48 h growth, the capillary reactor was inoculated with isolate 80 and the flow was stopped for 3 h to allow attachment. The bacterial biofilms were stained with rhodamine B (reference colour) and observed with CLSM 24 h after inoculation with isolate 80 (Fig. 4). Isolate *gfp*-17 was identified by green fluorescence due to the production of GFP, and isolate 80 was identified by rhodamine B. The excitation and emission wavelengths were distant between the fluorophores and did not overlap. Isolate *gfp*-17 established a green lawn that colonised the reactor surface, while isolate 80 was observed as spatially distributed red cell clumps within the established biofilm. Furthermore, cross sectional analysis of the biofilm (Fig. 5) showed that isolate 80 was not only attached to the surface of the isolate 17 biofilm, but that the cells were incorporated into the three dimensional structure of the established biofilm, suggesting that isolate 80 was able to migrate into the established biofim despite its lack of twitching and swimming motility.

## Discussion

The CF lung can be colonised by *P. aeruginosa *isolates that display heterogeneity in both motility and biofilm phenotype. We evaluated the association between types of motility and biofilm formation using a set of 96 clinical isolates of *P. aeruginosa*. Several studies have reported that motility is required to initiate cell attachment [8,37-39] although there is still no consensus as to the contribution of each type of motility to the overall process of biofilm development. While *P. aeruginosa *is a motile bacterium, the lack of motility in CF isolates has been previously reported [15] and here some 47% of the isolates were non-motile. Nonetheless, motility was not a key factor for the initiation of initial attachment *in vitro*, but when the motility phenotype was present, there was a notable increase in biofilm biomass similar to that observed by Head and Yu [40].

We were particularly interested in the role of Type IV pili in biofilm formation and we noted that our isolates had a broadly similar distribution of pilin types to that described by Klausen et al. [28], with no particular bias towards any TFP group for motile and non-motile isolates (Table 4). Some 65% of the isolates had Group I pilins, and although this group contained both motile and non-motile strains, we did however note a high degree of sequence diversity (data not shown), which could explain our observation that only 59% of *pilA*^+ ^isolates actually showed a twitching motility phenotype.

It is generally accepted that flagella are required for *P. aeruginosa *swimming and swarming motility [21,41]. We therefore deployed a combination of molecular and microscopic techniques to examine our selected isolates. As documented in the literature, however, the presence and expression of *fliC *was not enough to guarantee swimming motility [38,41,42], and our confirmation by SEM that certain non-swimming isolates possessed flagella leads to the hypothesis that other molecules must be involved in the initial colonisation of a surface by bacteria. Indeed, a recent study of *Staphylococcus epidermidis *biofilm identified a surface-associated autolysin that possessed bacteriolytic and adhesive properties [43] and it is possible that similar adhesins may play an important role in the initial attachment of *P. aeruginosa *to surfaces.

Differences in biofilm structure have been connected with the role of type IV pili and flagella [44] and in addition to diversity in biofilm biomass, we too observed variations in biofilm morphology amongst our isolates. Of the five isolates we investigated *in vitro*, only one formed the expected mushroom architecture, two failed to form a biofilm on the capillary (and were also only weakly attached in microtitre plate assays), one formed a thick lawn and one produced a thin lawn with hillocks. It is clear therefore that biofilm morphology and architecture are very isolate specific.

Bacterial immigration along a surface may be type IV pilus-driven [21] or flagellum-driven [22]. Klausen *et al*. [44] and Barken *et al*. [45] identified flat biofilm structures of both the parent PAO1 and the flagellum deficient mutant *ΔfliM-*PAO1, whilst the pilus deficient mutant *ΔpilA-*PAO1 formed hilly structures, suggesting that cell migration within the biofilm was the result of the type IV pili-driven motility. In contrast our experiments showed that twitching positive isolates produced a mushroom shaped biofilm or hillocks, whilst twitching negative isolates produced only thick lawns (Fig. 3).

Such diversity in the production, architecture and control of biofilm formation suggested to us that what we were measuring *in vitro *may not represent the true situation that would be found *in vivo*. For example, clinical isolates that do not produce biofilms in the laboratory must have the ability to survive in the patient lung, leading to the hypothesis that a synergy between isolates *in vivo*, may allow "non biofilm-forming" isolates to be incorporated into the biofilm.

Leriche *et al*. [19] have described the protection of certain bacterial strains by other strains within a mixed biofilm system. We therefore investigated the potential for a "non biofilm-forming" isolate (isolate 80) to be incorporated into the biofilm produced by isolate 17, a strong biofilm producer and showed that not only can an established biofilm of *P. aeruginosa *assist in the attachment and colonisation of another isolate, but also that the two *P. aeruginosa *isolates became integrated in a mixed biofilm as shown in cross section CSLM images (Fig. 5).

In the mixed biofilm scenario *in vitro*, the CF *P. aeruginosa *biofilm could consist of many different isolates, some of which are unable to form biofilms themselves yet can colonise an already established biofilm. Adaptability is the key to successful colonisation of an environmental niche and in the field of infectious disease, it is widely accepted that a pathogen will normally have more than one way of exerting a pathogenic effect. Many pathogens, therefore, have multiple adhesion mechanisms allowing attachment to, for example, epithelial cells [46]. We contend that the physiological mechanisms involved in biofilm formation should be considered in a similar manner, in that a deficiency in one phenotypic aspect of biofilm formation may be compensated for by other genetic and phenotypic factors.

## Conclusions

Motility makes a positive contribution to biofilm formation in CF isolates of *P. aeruginosa*, but is not an absolute requirement. It is clear that CF isolates with differing motility phenotypes can act synergistically to form a mixed biofilm. This could give an advantage to bacterial communities as they would possess a greater repertoire of genetic ability, thus allowing them to adapt to different challenges e.g. antibiotic chemotherapy, host inflammatory responses, etc.

## Authors' contributions

JSGD and JEM contributed to the design of the study, JEM and JSE arranged for provision of the *P. aeruginosa *CF strain collection and ED carried out the RAPD analysis, motility assays, microtitre plate analysis, gfp tagging, biofilm reactor work and all microscopy/image analysis. SP carried out detection of *pilA *and *fliC *genes and cloning and sequence analysis thereof, DB carried out the statistical analysis and ED, NGT, RWH and JSGD wrote the paper. All authors read and approved the final manuscript.
